# Silent victims: risk factors associated with school violence in Peruvian adolescents

**DOI:** 10.15649/cuidarte.4878

**Published:** 2025-10-07

**Authors:** Jhan Carlos Manuel Fernández-Delgado, Francisca Edita Diaz-Villanueva, Carlos Jesus Canova-Barrios, Felipe Machuca-Contreras, Maria Kappes, Eman Sameh AbdElhay

**Affiliations:** 1 School of Nursing, Universidad Nacional de Cajamarca, Cajamarca, Perú. E-mail: jfernandezd18_2@unc.edu.pe Universidad Nacional de Cajamarca Cajamarca Perú jfernandezd18_2@unc.edu.pe; 2 School of Nursing, Universidad Nacional de Cajamarca, Cajamarca, Perú. E-mail: ediazv@unc.edu.pe Universidad Nacional de Cajamarca Cajamarca Perú ediazv@unc.edu.pe; 3 Universidad Nacional del Oeste (UNO). Partido de Merlo, Argentina. E-mail: carlos.canova1993@gmail.com Universidad Nacional del Oeste (UNO) Partido de Merlo Argentina carlos.canova1993@gmail.com; 4 Universidad Autónoma de Chile. Santiago, Chile. E-mail: felipe.machuca@uautonoma.cl Universidad Autónoma de Chile Santiago Chile felipe.machuca@uautonoma.cl; 5 Faculty of Health Care Sciences. School of Nursing. Universidad San Sebastian, Puerto Montt, Chile. E-mail: maria.kappes@uss.cl Universidad San Sebastian Riyadh Chile maria.kappes@uss.cl; 6 Assistant Professor of Psychiatric Nursing and Mental Health, Faculty of Nursing, Mansoura University. Al-Mansurah, Egypt. E-mail: eman_2004@mans.edu.eg Mansoura University Al-Mansurah Egypt eman_2004@mans.edu.eg

**Keywords:** School Violence, Adolescent, Risk Factors, Violence, Violencia Escolar, Adolescente, Factores de Riesgo, Violencia, Violência Escolar, Adolescente, Fatores de Risco, Violência

## Abstract

**Introduction:**

School violence is a global and complex problem.

**Objective:**

Identify the types of school violence and their associated factors in Peruvian adolescents.

**Materials and Methods:**

An analytical cross-sectional study was conducted. Two self-administered instruments were administered to 253 adolescents selected through stratified random sampling from the first to fifth grade of secondary school at a Peruvian public institution in 2024. Bivariate and multivariate regression analyses were used to identify factors associated with school violence.

**Results:**

Verbal violence (63.24%) and psychological violence (54.94%) were the most prevalent, while physical violence (37.55%) and sexual violence (3.95%) were less frequent. The most influential factors were individual (75.49%), social (62.87%), and family (56.13%) factors, whereas community (35.56%), cultural (35.97%), and school (43.10%) factors had less influence. Bivariate analysis revealed significant associations between school violence and sex (p = 0.03), family type (p = 0.02), socioeconomic status (p = 0.01), and area of residence (p = 0.03). Multivariate analysis found an association between individual, family, and social factors and school violence, specifically with verbal and psychological violence.

**Discussion:**

These findings confirm the central role of personal, social, and family dynamics in shaping experiences of school violence. Addressing only school-related factors may be insufficient; interventions should also target adolescents' interpersonal environments to achieve long-term impact.

**Conclusion:**

School violence is a complex and multifactorial phenomenon. Comprehensive intervention strategies are recommended, not only to reduce violence but also to promote positive school climates that support learning and emotional well-being.

## Resumen

**Introducción:**

La violencia escolar es un problema global y complejo.

**Objetivo:**

Identificar los tipos de violencia escolar y sus factores asociados en adolescentes peruanos.

**Materiales y métodos:**

Estudio analítico transversal, que aplicó dos instrumentos autoadministrados a 253 adolescentes de manera aleatoria estratificada, desde primero hasta quinto grado de secundaria en una institución pública peruana en 2024. Se utilizaron análisis de regresión bivariados y multivariados para identificar factores asociados con violencia escolar.

**Resultados:**

La violencia verbal (63,24%) y la violencia psicológica (54,94%) fueron las más prevalentes mientras que la violencia física (37,55%) y la violencia sexual (3,95%) fueron menos frecuentes. Los factores más influyentes fueron los individuales (75,49%), sociales (62,87%) y familiares (56,13%), mientras que los de menor influencia fueron los comunitarios (35,56%), culturales (35,97%) y escolares (43,10%). El análisis bivariado mostró relaciones significativas entre la violencia escolar y el sexo (p = 0,03), el tipo de familia (p = 0,02), el nivel socioeconómico (p = 0,01) y el lugar de residencia (p = 0,03). El análisis multivariado encontró asociación entre los factores individuales, familiares y sociales con la violencia escolar, específicamente con la violencia verbal y la violencia psicológica.

**Discusión:**

Estos hallazgos confirman el papel central de las dinámicas personales, sociales y familiares en la configuración de las experiencias de violencia escolar. Abordar únicamente los aspectos institucionales puede resultar insuficiente; las intervenciones también deben centrarse en los entornos interpersonales de los adolescentes para lograr un impacto duradero.

**Conclusiones:**

La violencia escolar es un fenómeno multifactorial complejo, por lo que se recomiendan estrategias de intervención integrales que no solo trabajen en la reducción de la violencia, sino también en la promoción de climas escolares positivos que favorezcan el aprendizaje y el bienestar emocional.

## Resumo

**Introdução:**

A violência escolar é um problema global e complexo.

**Objetivo:**

Identificar os tipos de violência escolar e seus fatores associados em adolescentes peruanos.

**Materiais e métodos:**

Foi realizado um estudo transversal analítico. Dois instrumentos autoaplicáveis foram aplicados a 253 adolescentes selecionados por amostragem aleatória estratificada do primeiro ao quinto ano do ensino médio em uma instituição pública peruana em 2024. Análises de regressão bivariada e multivariada foram utilizadas para identificar os fatores associados à violência escolar.

**Resultados:**

A violência verbal (63,24%) e a violência psicológica (54,94%) foram as mais prevalentes, enquanto a violência física (37,55%) e a violência sexual (3,95%) foram menos frequentes. Os fatores mais influentes foram os individuais (75,49%), os sociais (62,87%) e os familiares (56,13%), enquanto os comunitários (35,56%), os culturais (35,97%) e os escolares (43,10%) tiveram menor influência. Análises bivariadas revelaram associações significativas entre violência escolar e sexo (p = 0,03), tipo de família (p = 0,02), status socioeconômico (p = 0,01) e área de residência (p = 0,03). A análise multivariada encontrou uma associação entre fatores individuais, familiares e sociais e violência escolar, especificamente com violência verbal e psicológica.

**Discussão:**

Essas descobertas confirmam o papel central das dinâmicas pessoais, sociais e familiares na formação de experiências de violência escolar. Abordar apenas fatores relacionados à escola pode ser insuficiente; as intervenções também devem ter como alvo os ambientes interpessoais dos adolescentes para alcançar impacto a longo prazo.

**Conclusões:**

A violência escolar é um fenômeno complexo e multifatorial. Estratégias abrangentes de intervenção são recomendadas, não apenas para reduzir a violência, mas também para promover climas escolares positivos que apoiem a aprendizagem e o bem-estar emocional.

## Introduction

School violence is a complex social and public health issue that encompasses various forms of repeated and intentional abuse—physical, verbal, psychological, and sexual^1^,^2^. Although it has long existed within academic settings, it has only recently received greater attention. Historically, the focus was placed almost exclusively on physical aggression, overlooking other harmful behaviors normalized in everyday interactions, such as jokes and insults^3^. These forms of violence have a profound impact on students’ emotional well-being, self-esteem, social development, and academic performance, with consequences that extend to family life, the school climate, and adolescent mental health—often triggering anxiety, depression, and risk behaviors^4^,^5^. Therefore, a comprehensive response is required from educational institutions, families, and society at large.

The high prevalence of school violence worldwide—affecting one in three students—highlights the importance of understanding its associated factors^6^. Identifying the types of violence and their determinants (individual, family, social, community, cultural, and school-related) is essential for designing effective prevention and intervention strategies. Each factor contributes differently to the type and frequency of violence experienced, underscoring the need for a holistic approach to foster safe, healthy educational environments^7^,^8^.

In response to this issue, the Peruvian Ministry of Education (MINEDU) launched the Specialized System for Reporting School Violence (SíseVe) in 2013. This platform allows confidential reporting of incidents through a website, email, phone, WhatsApp, or mobile application, and cases are followed up according to established protocols^9^. In 2024, SíseVe recorded more incidents in public schools (76%) than in private schools (24%), with girls (51%) more frequently affected than boys (49%). Adolescents were the most affected group (58%)^10^.

Despite policy advances—such as Law 31902 (2023), which requires the presence of at least one psychologist per school and the installation of video surveillance—implementation remains insufficient, with 98% of schools lacking these resources^11^. The persistence of school violence has led to the declaration of a national emergency regarding school coexistence, highlighting the urgent need for the effective enforcement of existing legal measures.

This study aimed to provide evidence on the magnitude and risk factors of school violence among Peruvian adolescents, thereby contributing to greater awareness and informed policy development. As the first study of its kind in Peru, it highlights the need for targeted prevention programs that may significantly reduce the long-term costs of unaddressed school violence. Its findings also offer insights to inform future research and interventions in educational and social policy. The aim of the study was to identify the types of school violence and their associated factors among Peruvian adolescents.

## Materials and Methods

**Study design and sample**

A cross-sectional, analytical study was conducted with a population of 732 secondary school students from a public institution in Peru. The study subjects were Peruvian adolescents aged 12 to 17 years. The sample size was calculated using a statistical formula for finite populations^12^ with a 95% confidence level and a 5% margin of error. Based on these parameters, 253 were included. A proportional stratified sampling technique was applied to determine the number of students per grade, from the first to the fifth year of secondary school. Inclusion criteria considered both sexes, parental consent for participation of their minor child, and the adolescent’s voluntary assent. Those participants who did not demonstrate sufficient commitment to completing the study instruments were excluded.

**Instruments**

The sociodemographic questionnaire collected data on sex, age, grade level, school shift, family type, socioeconomic status, and area of residence. The study instruments were developed and validated by the researchers through expert judgment by ten evaluators (four psychologists, three nurses, and three schoolteachers), who assessed clarity, organization, and relevance. Content validity indices showed an Aiken’s V of 0.98 for the school violence instrument and 1.0 for the instrument assessing risk factors for school violence. Internal consistency was high, with Kuder-Richardson coefficients of 0.89 and 0.86, respectively. Both instruments were pilot-tested with 25 participants with characteristics similar to those of the study sample.

The school violence instrument included four dimensions: psychological violence (isolation, control, contempt, distrust, humiliation, intimidation, and threats) with seven items; physical violence (beating, pushing, use of objects, violence disguised as playing around, attempted murder) with five items; verbal violence (insults, shouting, slander) with three items, and sexual violence (non-consensual touching, sexual coercion, sexual harassment, exposure to sexual material) with four items. Responses were dichotomous (Yes = 1, No = 0), for a total possible score of 19 points.

The risk factors for school violence instrument assessed six dimensions: individual (impulsiveness, poor skills, exposure to violence, self-control difficulties, victimization, gang involvement, substance use) with seven items; family (domestic violence, poor parental supervision, low emotional support, verbal conflicts) with four items; social (peer pressure, participation in violence, misunderstandings) with three items; school-related (absence of rules, low emotional support, negative climate) with three items; cultural (discrimination, machismo, aggressive role models) with three items, and community (crime, lack of recreational resources, poor cohesion) with three items. All responses were dichotomous (Yes = 1, No = 0), for a total score of 22 points.

**Data collection**

Two nurses were trained to administer the instruments. Parents or legal guardians were informed about the study objective and ethical principles. The support of the school principal facilitated engagement. She first called meetings with the parents or guardians of the randomly selected students. The meetings were organized by academic year and held on different days in September 2024. Before data collection, information sessions were conducted with the students to explain the types of violence and risk factors included in the instruments, ensuring that the concepts were clearly understood by the participants. Recruitment and data collection took place between October and December 2024.

**Data analysis**

**Figure 1:**
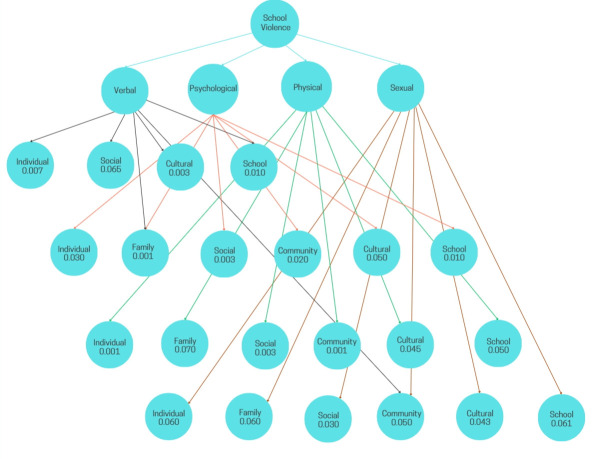
Relationship between types of school violence and risk factors in Peruvian adolescents

*Note: p <0.05 indicates a significant relationship.*

Data were processed using IBM SPSS Statistics, version 27. Frequencies and percentages were calculated for each indicator. The Chi-square test of Pearson was used to analyze the relationship between school violence and sociodemographic variables; when the assumptions of the Chi-square test were not met (expected cell counts <5), Fisher’s exact test was applied. A significance level of p <0.05 was considered. Bivariate logistic regression analysis was performed to estimate crude odds ratios (COR) with 95% confidence intervals (CI). Subsequently, a multivariate analysis was conducted using a binomial regression model to estimate adjusted odds ratios (AOR) with 95% CI. Model fit was assessed using the Hosmer–Lemeshow goodness-of-fit test to verify the agreement between observed data and model-predicted values. Figure 1was created using Moqups (Evercoder Software SRL).

**Availability of data and materials**

The dataset used in this research is available in a Mendeley repository^13^.

**Ethical considerations**

The research followed the international ethical principles outlined in the Declaration of Helsinki and complied with Supreme Decree No. 021-2017-SA of the Peruvian Ministry of Health, which establishes guidelines for health research involving minors. Informed consent was obtained from parents or legal guardians, and informed assent was obtained from all participants. The principles of autonomy, justice, and non-maleficence were upheld. The study was approved by resolution of the Nursing Faculty Council at Universidad Nacional de Cajamarca.

## Results

**Sociodemographic information**

The students’ ages ranged from 12 to 17 years, with females representing 58.10% and males 41.90% of the sample. Educational levels in secondary school extended over five years: first year (20.17%), second year (20.17%), third year (19.32%), fourth year (20.17%), and fifth year (20.17%). The school shift was morning for 44.66% and afternoon for 55.34%. Regarding family type, 22.53% lived in nuclear families, 43.08% in single-parent families, and 34.39% in extended families. Socioeconomic status was characterized as poverty (43.87%), extreme poverty (15.42%), and non-poor (40.71%). The area of residence was urban in 53.36% and rural in 46.64% of the sample. Bivariate analysis identified significant associations between school violence and sex (p = 0.03), family type (p = 0.02), socioeconomic status (p = 0.01), and area of residence (p = 0.03). (See Table 1).

**Table 1:** Characteristics of participants involved in school violence (n=253)

Sociodemographic variables	Total % (n)	School violence		p-value
		Yes % (n)	No % (n)	
Sex				**0.030^a^**
Male	41.90 (106)	67.90 (72)	32.10(34)	
Female	58.10 (147)	34.7 (51)	65.3 (96)	
Age (M: 14.07, SD: 1.44)				**0.120^b^**
12 years	18.18 (46)	19.60 (9)	80.40 (37)	
13 years	20.94 (53)	22.60 (12)	77.40 (41)	
14 years	20.17 (51)	35.30 (18)	64.70 (33)	
15 years	18.58 (47)	36.20 (17)	63.80 (30)	
16 years	20.94 (53)	26.40 (14)	73.60 (39)	
17 years	1,19 (3)	33.30 (1)	66.70 (2)	
Secondary school year				**0.180^a^**
First	20.17 (51)	23.50 (12)	76.50 (39)	
Second	20.17 (51)	19.60 (10)	80.40 (41)	
Third	19.32 (49)	34.70 (17)	65.30 (32)	
Fourth	20.17 (51)	31.40 (16)	68.60 (35)	
Fifth	20.17 (51)	29.40 (15)	70.60 (36)	
School shift				**0.100^a^**
Morning	44.66 (113)	32.70 (37)	67.30 (76)	
Afternoon	55.34 (140)	23.60 (33)	76.40 (107)	
Family type				**0.020^a^**
Nuclear	22.53 (57)	38.60 (22)	61.40 (35)	
Single-parent	43.08 (109)	33.03 (36)	66.97 (73)	
Extended	34.39 (87)	13.79 (12)	86.21 (75)	
Socioeconomic status				**0.010^a^**
Non-poor	40.71 (103)	35.92 (37)	64.08 (66)	
Poor	43.87 (111)	18.02 (20)	91.98 (91)	
Extreme poverty	15.42 (39)	33.33 (13)	66.67 (26)	
Area of residence				**0.030^a^**
Urban	53.36 (135)	30.37 (41)	69.63 (94)	
Rural	46.64 (118)	24.58 (29)	75.42 (89)	

95% CI: 95% Confidence Interval. p <0.05 indicates a statistically significant association with school violence. ^a^p calculated using Pearson’s Chi-square test. ^b^p calculated using Fisher’s exact test.

**Types of school violence in adolescents**

Four types of school violence were reported. In the psychological dimension, 54.94% of adolescents were affected, with a COR of 2.00 (95% CI: 1.52-2.35) and an AOR of 2.00 (95% CI: 1.52-2.12), confirming that adolescents experience this type of school violence. The most frequent subtypes were isolation and humiliation (67.19%). Logistic regression analysis showed a COR of 2.52 (95% CI: 2.24-2.90) and an AOR of 2.42 (95% CI: 2.00-2.82), confirming a high probability of exposure to these subtypes of violence in similar contexts.

In the physical dimension, 37.55% of adolescents were affected, with a COR of 1.52 (95% CI: 1.25- 1.82) and an AOR of 1.42 (95% CI: 1.14-1.72). Beating was reported by 30.83% (COR = 1.25 [95% CI: 1.05-1.52]; AOR = 1.12 [95% CI: 0.90-1.43]) and pushing by 28.85% (COR = 1.22 [95% CI: 1.00-1.50]; AOR = 1.10 [95% CI: 0.90-1.4]).

The verbal dimension presented the highest prevalence, affecting 63.24% of adolescents, with a COR of 2.62 (95% CI: 2.33-3.05) and an AOR of 2.32 (95% CI: 1.92-2.72). Insults were reported by 73.52% of participants, with a COR of 3.15 (95% CI: 2.75-3.68) and an AOR of 3.00 (95% CI: 2.61-3.25), highlighting this subtype as a key indicator of verbal violence.

In the sexual dimension, 3.95% of adolescents reported being affected, representing the lowest prevalence. Logistic regression showed a COR of 0.20 (95% CI: 0.12-0.35) and an AOR of 2.32 (95% CI: 1.92-2.72). Exposure to sexual material was the most frequent subtype (4.74%), with a COR of 0.27 (95% CI: 0.16-0.32) and an AOR of 0.25 (95% CI: 0.11-0.32). (See Table 2)

**Table 2:** Types of school violence experienced by Peruvian adolescents (n=253)

Dimensions	Yes % (n)	No % (n)	COR (95% CI)	AOR (95% CI)	p-value
Psychological violence	**54.94 (139)**	**45.06 (114)**	2.00(1.52-2.35)	2.00(1.52-2.12)	**0.003**
Isolation	67.19 (170)	32.81 (83)	2.52(2.24-2.90)	2.42(2.00-2.82)	<0.001
Control	47.04 (119)	52.96 (134)	1.81(1.52-2.22)	1.67(1.32-2.00)	0.010
Contempt	42.29 (107)	57.71 (146)	1.65(1.35-2.00)	1.51(1.25-1.81)	0.005
Distrust	52.17 (132)	47.83 (121)	2.00(1.70-2.42)	1.80(1.56-2.12)	0.002
Humiliation	67.19 (170)	32.81 (83)	2.52(2.24-2.90)	2.42(2.00-2.82)	0.001
Intimidation	35.97 (91)	64.03 (162)	1.32(1.00-1.75)	1.24(1.00-1.52)	0.050
Threats	48.22 (122)	51.78 (131)	2.00(1.72-2.34)	1.81(1.53-2.10)	0.010
Physical violence	**37.55 (95)**	**62.45 (158)**	1.52(1.25-1.82)	1.42(1.14-1.72)	0.016
Beating	30.83 (78)	69.17 (175)	1.25(1.05-1.52)	1.12(0.90-1.43)	0.060
Pushing	28.85 (73)	71.15 (180)	1.22(1.00-1.50)	1.10(0.90-1.4)	0.055
Using objects	26.88 (68)	73.12 (185)	1.05(0.95-1.42)	1.05(0.8-1.3)	0.070
Violence disguised as playing around	16.60 (42)	83.40 (211)	0.82(0.67-1.05)	0.72(0.5-0.9)	0.020
Attempted murder	0.40 (1)	99.60 (252)	0.10(0.00-0.10)	0.13(0.0-0.1)	0.001
Verbal violence	**63.24 (160) **	**36.76 (93)**	2.62(2.33-3.05)	2.32(1.92-2.72)	0.012
Insults	73.52 (186)	26.48 (67)	3.15(2.75-3.68)	3.00(2.61-3.25)	0.002
Shouting	48.22 (122)	51.78 (131)	2.00(1.72-2.32)	1.85(1.52-2.17)	0.026
Slander	35.97 (91)	64.03 (162)	1.37(1.00-1.75)	1.23(1.00-1.52)	0.050
Sexual violence	** 3.95 (10)**	**96.05 (243)**	0.20(0.12-0.35)	0.20(0.10-0.35)	0.001
Non-consensual touching	0.40 (1)	99.60 (252)	0.12(0.05-0.13)	0.10(0.10-0.12)	<0.001
Sexual coercion	1.19 (3)	98.81 (250)	0.15(0.00-0.20)	0.11(0.04-0.26)	<0.001
Sexual harassment	1.19 (3)	98.81 (250)	0.12(0.00-0.25)	0.10(0.00-0.20)	<0.001
Exposure to sexual material	4.74 (12)	95.26 (241)	0.27(0.16-0.32)	0.25(0.11-0.32)	0.001

COR: Crude odds ratio; AOR: adjusted odds ratio; 95% CI: 95% Confidence interval. COR values > 1 indicate higher crude odds of experiencing the corresponding type or subtype of school violence. AOR values > 1 indicate higher adjusted odds. Model fit was verified using the Hosmer-Lemeshow test (p = 0.403), confirming adequate model fit.

**Risk factors for school violence**

Six categories of risk factors associated with school violence were identified. The individual factors were reported by 75.49% of participants, with a COR of 3.00 (95% CI: 2.62-4.02) and an AOR of 3.00 (95% CI: 2.61-3.75), indicating a greater probability of occurrence and bold associations (p = 0.001). The logistic regression analysis showed that impulsiveness (66.80%; COR = 2.41 [95% CI: 1.60-3.42]; AOR = 2.10 [95% CI: 1.42-3.21]; p = 0.003) and poor skills (64.03%; COR = 2.25 [95% CI: 1.51-3.32]; AOR = 2.00 [95% CI: 1.30-3.00]; p = 0.005) are the most reported subfactors in this dimension with significant associations with school violence.

The family factors were reported by 56.13% of participants, with a COR of 2.62 (95% CI: 1.42-3.10), an AOR of 2.50 (95% CI: 1.30-2.81), and p-value of 0.007. Low emotional support was reported by 67.55%, with a COR of 2.61 (95% CI: 1.81-3.83), an AOR of 2.35 (95% CI: 1.66-3.51), and p-value of 0.001, indicating a bold association with school violence.

The social factors were reported by 62.87% of the students, with a COR of 2.30 (95% CI: 1.63-3.35), an AOR of 2.00 (95% CI: 1.42-3.15), and p-value of 0.001. Participation in violence (59.29%; COR = 2.21 [95% CI: 1.50-3.20]; AOR = 2.00 [95% CI: 1.30-3.00]; p = 0.014) and misunderstandings (57.71%; COR = 2.00 [95% CI: 1.41-2.92]; AOR = 1.72 [95% CI: 1.27-2.62]; p = 0.001) were the most influential subfactors.

The school-related factors were reported by 43.10% of participants, with a COR of 1.75 (95% CI: 1.25- 2.63), an AOR of 1.52 (95% CI: 1.11-2.34), and p-value of 0.060. Low emotional support was the main subfactor (43.87%) with a COR of 1.95 (95% CI: 1.31-2.82), an AOR of 1.62 (95% CI: 1.11-2.41), and p-value of 0.050.

The cultural factors were reported by 35.97% of the participants, with a COR of 1.62 (95% CI: 1.10-2.52), an AOR of 1.40 (95% CI: 0.95-2.12), and p-value of 0.050. Machismo was the predominant subfactor (50.59%) with a COR of 2.00 (95% CI: 1.45-3.00), an AOR of 1.85 (95% CI: 1.33-2.77), and p-value of 0.003.

The community factors were reported by 35.56% of the students, with a COR of 2.00 (95% CI: 1.41-3.10), an AOR of 1.82 (95% CI: 1.32-2.85), and p-value 0.050. Lack of recreational resources (37.13%) was the most frequently reported subfactor, with a COR of 1.51 (95% CI: 1.00-2.36), an AOR of 1.50 (95% CI: 1.00-2.31), and p-value 0.150. (See Table 3)

**Table 3:** Risk factors associated with school violence in Peruvian adolescents (n=253)

Dimensions	Yes % (n)	No % (n)	COR (95% CI)	AOR (95% CI)	p-value
Individual factors	**75.49 (191)**	**24.51 (62)**	3.00(2.62-4.02)	3 .00(2.61-3.75)	**0.001**
Impulsiveness	66.80 (169)	33.20 (84)	2.41(1.60-3.42)	2.10(1.42-3.21)	0.003
Poor skills	64.03 (162)	35.97 (91)	2.25(1.51-3.32)	2.00(1.30-3.00)	0.005
Exposure to violence	54.94 (139)	45.06 (114)	1.82(1.25-2.60)	1.65(1.11-2.42)	0.015
Self-control difficulties	57.71 (146)	42.29 (107)	2.00(1.43-2.90)	1.71(1.24-2.66)	0.010
Victimization	35.16 (89)	64.84 (164)	1.37(0.90-1.82)	1.20(0.80-1.75)	0.020
Gang involvement	3.16 (8)	96.84 (245)	1.10(0.45-3.23)	0.9(0.31-2.73)	0.150
Substance use	4.74 (12)	95.26 (241)	1.12(0.44-3.20)	1.0(0.36-3.01)	0.650
Family factors	**56.13 (142)**	**43.87 (111)**	2.62(1.42-3.10)	2.50(1.30-2.81)	**0.007**
Domestic violence	20.17 (51)	79.83 (202)	1.51(0.94-2.61)	1.43(0.83-2.42)	0.110
Poor parental supervision	45.87 (116)	54.13 (137)	1.70(1.25-2.50)	1.52(1.00-2.33)	0.020
Low emotional support	67.55 (171)	32.45 (82)	2.61(1.81-3.83)	2.35(1.66-3.51)	0.001
Verbal conflicts	43.87 (111)	56.13 (142)	1.90(1.30-2.81)	1.67(1.15-2.48)	0.002
Social factors	**62.87 (159)**	**37.13 (94)**	2.30(1.63-3.35)	2.00(1.42-3.15)	**0.001**
Peer pressure	42.68 (108)	57.32 (145)	1.81(1.24-2.75)	1.65(1.16-2.45)	0.016
Participation in violence	59.29 (150)	40.71 (103)	2.21(1.50-3.20)	2.00(1.30-3.00)	0.014
Misunderstandings	57.71 (146)	42.29 (107)	2.00(1.41-2.92)	1.72(1.27-2.62)	0.001
School-related factors	**43.10 (109)**	**56.90 (144)**	1.75(1.25-2.63)	1.52(1.11-2.34)	0.060
Absence of rules	32.45 (82)	67.55 (171)	1.51(1.00-2.37)	1.30(0.95-2.00)	0.040
Low emotional support	43.87 (111)	56.13 (142)	1.95(1.31-2.82)	1.62(1.11-2.41)	0.050
Negative climate	26.88 (68)	73.12 (185)	1.46(0.90-2.22)	1.27(0.82-1.90)	0.040
Cultural factors	**35.97 (91)**	**64.03 (162)**	1.62(1.10-2.52)	1.40(0.95-2.12)	0.050
Discrimination	26.88 (68)	73.12 (185)	1.47(0.93-2.00)	1.21(0.80-1.84)	0.062
Machismo	50.59 (128)	49.41 (125)	2.00(1.45-3.00)	1.85(1.33-2.77)	0.003
Aggressive role models	28.85 (73)	71.15 (180)	1.56(1.00-2.36)	1.30(0.98-2.03)	0.015
Community factors	**35.56 (90)**	**64.44 (163)**	2.00(1.41-3.10)	1.82(1.32-2.85)	0.050
Crime	34.84 (88)	65.16 (165)	2.33(1.62-3.54)	2.30(1.60-3.50)	0.020
Lack of recreational resources	37.13 (94)	62.87 (159)	1.51(1.00-2.36)	1.50(1.00-2.31)	0.150
Poor cohesion	28.47 (72)	71.53 (181)	1.45(0.92-2.10)	1.39(0.96-2.00)	0.650

COR = Crude odds ratio; AOR = Adjusted odds ratio; 95% CI = 95 % Confidence interval. COR values > 1 indicate higher crude odds of experiencing the corresponding risk factor; AOR values > 1 indicate higher adjusted odds. p <0.05 indicates a statistically significant association with school violence. Model fit was confirmed using the Hosmer–Lemeshow test (p = 0.463).

**School violence and associated risk factors in adolescents**

Building on the previous findings, various types of school violence were significantly associated with different contextual factors. Verbal violence was associated with individual (p = 0.007), family (p = 0.001), cultural (p = 0.003), and school-related (p = 0.010) factors. Psychological violence was linked to individual (p = 0.030), family (p = 0.001), social (p = 0.003), community (p = 0.020), and school- related (p = 0.020) factors. Physical violence showed associations with individual (p = 0.001), social (p = 0.002), and community (p = 0.045) factors. Lastly, sexual violence was associated with social (p = 0.030) and cultural (p = 0.043) factors. (See Figure 1).

## Discussion

This study found a notable prevalence of verbal and psychological violence, affecting more than half of the participants, consistent with previous research^14^. Gutiérrez^15^ identified verbal violence as the most frequent type of violence (M=14.56, SD:2.46) in school contexts, especially in secondary school, attributing its persistence to social normalization and the difficulty of early detection. By contrast, López-Arancibia^16^ reported verbal violence in only 12% of the students, classifying it at a high level.

Guevara-Vidalón et al.^17^ identified psychological violence in 59.8% of the students and emphasized its lasting emotional impact, which affects academic performance and adolescent mental health. Although it showed a lower prevalence in this study, prior research by Cid et al.^18^ and Cedeño-Sandoya^19^ showed the relevance of physical and sexual violence and highlighted their critical impact, especially on the psychosocial well-being of victims, emphasizing the importance of prevention strategies.

The analysis of factors associated with school violence revealed that individual, family, and social dimensions play a key role. However, this study did not explore the interaction between these variables or whether specific combinations might amplify or buffer the risk of school violence. Future research should examine these interaction effects to gain a deeper understanding of the complex mechanisms underlying school violence. This finding is consistent with that of Cid et al.^18^, who noted that impulsiveness, poor parental supervision, and lack of social skills increase vulnerability to school violence. Similarly, Faria and Martins^20^ emphasized the role of family and social environments— particularly authoritarian or neglectful parenting styles and disorganized settings—as contributors to school violence. The results also align with Caracas-Moreira et al.^21^, who found that peer networks and social exclusion are critical factors in sustaining violent behaviors in schools.

Regarding the role of community, cultural, and school-related factors, their more limited influence alignswith García-Montañezand Ascensio-Martínez^22^, whoarguethatthesefactorshavecomparatively less impact on adolescents' experiences of school violence. These findings suggest that individual, family, and social factors play a more significant role in both perpetration and victimization, and that violence in schools should not be viewed as generated exclusively within the school environment. In this research, although the adjusted analysis showed an increased probability of school violence when considering these factors (AOR > 1), the lack of statistical significance suggests insufficient evidence to support a direct association in the bivariate analysis. This finding implies that other factors may be influencing the relationship observed in the adjusted model. Hamodi-Galán and Jiménez-Robles^23^ argue that school and community culture play a relevant role in preventing bullying, which could be an area for improvement in the Peruvian context.

Implementing school policies that reinforce peaceful coexistence and tolerance can help mitigate these problems, as highlighted by the United Nations Educational, Scientific and Cultural Organization (UNESCO)^24^ as a key component of effective prevention programs. The Kiva program in Finland is a successful example of how school culture can influence bullying prevention, recognizing it as a social problem that requires a response at the community level^25^.

In Peru, however, geographical and cultural diversity pose challenges for implementing standardized school-level interventions. Various authors emphasize the need to implement comprehensive strategies to address scholar violence in the country. Castillo-Pulido^26^ argues that bullying is a changing and difficult-to-solve phenomenon that cannot be solved only within the school environment. An ecological approach encompassing personal, family, and community factors is therefore required. This research supports this perspective, considering other factors and emphasizing the need for comprehensive programs that not only reduce violence but also promote learning and emotional well-being.

From a methodological point of view, this study is among the first in Peru to combine complementary statistical approaches, thereby producing more robust results. In particular, factor scores were estimated for each dimension using confirmatory factor analysis.

**Limitations**

This research on school violence among Peruvian adolescents has several limitations that warrant consideration. First, the research relied on self-reported data, which may be subject to biases such as underreporting or overreporting due to fear of judgment or social stigma. Adolescents may also lack the ability to adequately comprehend or express the extent of their experiences with violence, leading to incomplete responses. These limitations may impact the accuracy of the findings and the generalizability of the results.

Another limitation is the research's cross-sectional design, which provides only a snapshot of school violence and its associated factors at a specific point in time. This design restricts the capacity to establish causal relationships between individual, social, and family-level influences and the incidence of violence. Longitudinal studies would be more effective in monitoring temporal changes and elucidating how these factors contribute to the development and escalation of violence among adolescents.

Finally, this research focused only on Peruvian adolescents, limiting the applicability of its results to other cultural and geographical settings. The findings may not comprehensively reflect the experiences of adolescents in other nations or regions with different sociocultural frameworks or resource availability. Consequently, further research in diverse contexts is essential to validate and expand on these results.

**Implications**

The study's results underscore the need for nurses to implement routine screening for violence among adolescents, especially within educational environments. Given the predominance of verbal and psychological violence, nurses may significantly contribute to identifying at-risk or affected students by including questions on mental health and safety in standard health assessments. Early identification enables timely interventions and opens opportunities for assistance, including counseling and the development of coping skills. Nurses must adopt a holistic approach, taking into account not just physical health but also the psychological and social well-being of adolescents.

Since family and social factors substantially influence adolescent experiences of violence, nurses should work closely with families and communities to help mitigate these risk factors. Family relationships, social contexts, and socioeconomic conditions all influence the incidence of violence. By collaborating with social workers and counselors, nurses can assist adolescents in addressing these difficulties. It is equally important for nurses to recognize the gender-specific characteristics that may influence violence and to tailor interventions that meet adolescents' needs, regardless of gender. This may include offering gender-sensitive counseling and promoting open dialogue around gender-based violence.

Collaboration with educational institutions and communities is another key consideration for nursing practice. Nurses should partner with schools to design and implement violence prevention programs, such as anti-bullying campaigns and mental health awareness projects. Nurses can contribute to reducing school violence by fostering safe school environments and providing support services to students and educators. Furthermore, nurses should advocate for policies that protect adolescents and guarantee that schools are equipped with the necessary resources, including counselors and safe spaces, to mitigate the effects of violence and foster a supportive educational setting for all students.

## Conclusions

Verbal and psychological violence were the most common forms of school violence, whereas physical and sexual violence were less prevalent. This reflects a problem centered on less visible but equally harmful forms of aggression. Individual, social, and family factors were the most influential, showing that school violence cannot be attributed solely to school-related factors but rather results from a complex interaction of factors. In contrast, community, cultural, and school-related factors showed lower prevalence, suggesting the need for comprehensive strategies that address all levels of students' environment.

In this regard, future interventions should consider strengthening the socio-emotional approach within the school curriculum and fostering closer integration with community-based mental health services. Such measures could enhance early identification, prevention, and management of school violence in the Peruvian context.

Future research should explore longitudinal designs to examine how these factors evolve over time, or conduct regional comparative studies to identify local variations and inform the development of context-specific interventions.

## References

[1] .

[2] .

[3] .

[4] .

[5] .

[6] .

[7] .

[8] .

[9] .

[10] .

[11] .

[12] .

[13] .

[14] .

[15] .

[16] .

[17] .

[18] .

[19] .

[20] .

[21] .

[22] .

[23] .

[24] .

[25] .

[26] .

